# Symptom impact and health-related quality of life (HRQoL) assessment by cancer stage: a narrative literature review

**DOI:** 10.1186/s12885-024-12612-z

**Published:** 2024-07-22

**Authors:** Karen C. Chung, Anushini Muthutantri, Grace G. Goldsmith, Megan R. Watts, Audrey E. Brown, Donald L. Patrick

**Affiliations:** 1 GRAIL, Inc., 1525 O’Brien Dr, Menlo Park, CA 94025 USA; 2 Genesis Research Group, West One, Forth Banks, Newcastle upon Tyne, NE1 3PA UK; 3 University of Washington, 1959 NE Pacific St, Box 357660, Seattle, WA 98195 USA

**Keywords:** Cancer, Patient reported outcomes, Health related quality of life, Early detection, Cancer screening

## Abstract

**Background:**

Cancer stage at diagnosis is an important prognostic indicator for patient outcomes, with detection at later stages associated with increased mortality and morbidity. The impact of cancer stage on patient-reported outcomes is poorly understood. This research aimed to understand symptom burden and health related quality of life (HRQoL) impact by cancer stage for ten cancer types: 1) ovarian, 2) lung, 3) pancreatic, 4) esophageal, 5) stomach, 6) head and neck, 7) colorectal, 8) anal, 9) cervical, and 10) liver and bile duct.

**Methods:**

Ten narrative literature reviews were performed to identify and collate published literature on patient burden at different stages of disease progression. Literature searches were conducted using an AI-assisted platform to identify relevant articles published in the last five (2017–2022) or ten years (2012–2022) where articles were limited. Conference abstracts were searched for the last two years (2020–2022). The geographic scope was limited to the United States, Canada, Europe, and global studies, and only journal articles written in English were included.

**Results:**

A total of 26 studies with results stratified by cancer stage at diagnosis (and before treatment) were selected for the cancer types of lung, pancreatic, esophageal, stomach, head and neck, colorectal, anal, and cervical cancers. Two cancer types, ovarian cancer, and liver and bile duct cancer did not return any search results with outcomes stratified by disease stage. A general trend was observed for worse patient-reported outcomes in patients with cancer diagnosed at an advanced stage of disease compared with diagnosis at an earlier stage. Advanced disease stage was associated with greater symptom impact including general physical impairments such as pain, fatigue, and interference with functioning, as well as disease/region-specific symptom burden. Poorer HRQoL was also associated with advanced disease with commonly reported symptoms including anxiety and depression.

**Conclusions:**

Overall, the general trend for greater symptom burden and poorer HRQoL seen in late stage versus early-stage disease across the included cancer types supports the importance for early diagnosis and treatment to improve patient survival and decrease negative impacts on disease burden and HRQoL.

## Introduction

Cancer stage at diagnosis is an important prognostic indicator for patient outcomes, with detection at later stages associated with increased mortality and morbidity. An estimated 2,001,140 new cancer cases will occur in the United States in 2024 along with 611,720 cancer deaths [1]. Many cancers are diagnosed during late (distant) stage including 55% of ovarian cancer cases, 53% of lung cancer cases, 51% of pancreatic cancer cases, 38% of esophageal cancer cases, and 36% of stomach cancer cases [2]. Stage at diagnosis is an important predictor both for treatment efficacy and survival, but diagnosis times vary by cancer type [2].

The World Health Organization identified two strategies which allow for more effective cancer treatment: 1) diagnosing symptomatic cancer as early as possible, and 2) screening for asymptomatic cancer or pre-cancerous lesions in non-symptomatic target populations [3]. The US Preventive Services Task Force (USPSTF) recommends single cancer screening based on age and sex for lung (also risk-based), colon, and cervical cancers, among others [4–8]. For cancers with screening paradigms such as cervical cancer, most cases (43%) are diagnosed in early stage where 5-year survival rates are high (91.2%). However, for cancers without screening paradigms, such as pancreatic cancer, most cases (51%) are diagnosed in late stage, where 5-year survival rates are very low (3.2%) [2].

Currently, the level of invasiveness of cancer screening varies by cancer type and location, ranging from more invasive procedures such as colonoscopy for colorectal cancer and Papanicolaou test (pap smear) for cervical cancer, to less invasive procedures such as blood-based tests or imaging tests such as mammography or low-dose computed tomography. A key focus of current research for cancer screening is less invasive multi-cancer screening technologies, such as blood-based multi-cancer detection screening assays [9]. This multi-cancer early detection (MCED) approach has the potential to improve treatment outcomes through earlier diagnosis of a wide range of cancer types, in addition to improving health-related quality of life (HRQoL) for patients with a positive diagnosis.

Cancer type and cancer stage may be associated with specific symptomatology, with overall symptoms and symptom impact being greater in advanced disease stages [10]. Advanced cancer stage and increased symptoms are associated with worse HRQoL, which may be evaluated through patient reported outcome measures (PROMs). PROMs are self-reported questionnaires which provide a qualitative/quantitative measurement of various aspects of a patient’s health, including HRQoL, functional status, and symptoms and symptom burden, directly by the patient without clinician interpretation [11]. Inclusion of PROMs in clinical practice in oncology can be associated with benefits including improvements in care, prognosis, communication, patient safety, and risk identification such as symptom control and identification [12].

The purpose of this narrative review was to identify and collate published literature on symptom impact at different stages of disease progression for a range of 10 cancer types: 1) ovarian, 2) lung, 3) pancreatic, 4) esophageal, 5) stomach, 6) head and neck, 7) colorectal, 8) anal, 9) cervical, and 10) liver and bile duct. This will provide valuable information on symptom impact and HRQoL by cancer type and stage at diagnosis. In contrast to a systematic review, this narrative review was not intended to identify and report all the literature available for symptom impact but rather focused on the information most relevant to healthcare providers who are interested in understanding the burden of disease on patients with specific types of cancer.

## Methods

### Search strategy

Ten narrative literature reviews were performed to identify and collate published literature on patient burden at different stages of disease progression for the following cancer types: 1) ovarian, 2) lung, 3) pancreatic, 4) esophageal, 5) stomach, 6) head and neck, 7) colorectal, 8) anal, 9) cervical, and 10) liver and bile duct. Disease terms for each cancer type were run in an artificial intelligence (AI)-assisted platform (EVID PRO) [13] to identify relevant articles published within the last 5 years (January 2017—December 2022). Where the number of articles identified for a specific cancer type were limited to less than 10 articles, in the case of ovarian, esophageal, stomach, anal, cervical, and liver and bile duct cancers, this was extended to 10 years (January 2012—December 2022). The geographic scope was limited to the United States (U.S.), Canada, Europe and global studies, and only journal articles written in English were included. The EVID PRO tool automatically scans and pulls articles with any specific acronyms, scales, and/or PRO instruments. Electronic searches were supplemented with grey literature searches of relevant conference meeting abstracts restricted to the last 2 years (2020–2022). Congresses included: American Society of Clinical Oncology (ASCO), European Society for Medical Oncology (ESMO), and Professional Society for Health Economics and Outcomes Research (ISPOR) (for all indications), Digestive Disease Week (DDW) (GI cancers only), European Respiratory Society (ERS) (lung cancer), and American Head and Neck Society (AHNS) (head and neck).

### Study selection

For each literature review, an initial screening was performed on the title and abstract of the identified articles followed by a full-text review of articles considered relevant. The PICOS (Population, Intervention, Comparison, Outcomes, Study Design) criteria are shown in Table 1. Studies were considered for inclusion if patient staging information was reported; outcomes included impact of cancer-related symptoms by cancer type (by stage of disease preferred), as assessed by standardized/ validated instruments (e.g., EORTC QLQ-C30, MDASI); and met the geographic and language limits described above. Studies were excluded if the study population was pretreated (treatment may affect HRQoL/PRO and thus not reflect cancer specific symptoms/impact), if studies were conducted outside US/Europe (unless global), if outcomes reported were related to treatment or were only reported post intervention (no baseline scores), and if only instruments not previously validated were implemented.

**Table 1:** PICOS criteria

Element	Focus
Patients	Patients with staged cancer (e.g., AJCC): • Ovarian • Lung • Pancreatic • Esophageal • Stomach • Head and neck • Colorectal • Anal • Cervical • Liver/ bile duct Where a limited number of publications have been identified that include information on staging, other studies will be considered
Intervention/ comparator	• Any
Outcomes	• Severity and impact of cancer-related symptoms (e.g., pain, fatigue) by cancer type and stage, as assessed by standardized/ validated instruments (e.g., MDASI, EORTC-QLQ C30) • HRQoL/ PROs
Study types	• Any
Timeframe	• Literature published in the past five years. If limited literature is identified, time limit will be expanded to ten years
Geographic scope and language	• United States (US), Europe, Canada, and global studies • English language abstracts
Databases to search	• PubMed via Evid • Hand searches • American Society of Clinical Oncology (ASCO) • European Society for Medical Oncology (ESMO) • Digestive Disease Week (DDW); GI cancers • European Respiratory Society (ERS) and the American Thoracic Society (ATS); Lung cancer • Other conferences as appropriate for each oncology indication

### Data extraction

Following selection of relevant literature from screening and full text review, data from publications meeting the PICOS elements were extracted into standardized extraction tables in an Excel workbook by one reviewer. A second author reviewed all data extractions for completeness and accuracy. Any discrepancies encountered were discussed and resolved by a third independent reviewer.

## Results

Across all cancer types, 150 articles reporting PRO data that included information on disease staging were selected. In most of these studies PRO data were not reported with results stratified by disease stage, and instead reported outcomes with patients of varying disease stages grouped together (*n* = 54) or included patients within a particular disease stage (*n* = 70). After excluding these studies, 26 studies across 8 cancer types reporting PRO results stratified by disease stage were selected for inclusion. Two cancer types, liver and bile duct, and ovarian did not return any search results with outcomes stratified by disease stage.

Descriptions of the PRO instruments used in the 26 included studies are provided in Table 2 [14–33]. For each study across the 8 cancer types with results stratified by disease stage, a description including study design, PRO instruments and results, and any statistical analyses performed is presented in Table 3. The results for these 8 cancer types are organized by the primary stage at which each cancer type is most often diagnosed according to the National Cancer Institute SEER statistics: 1) late stage/distant, 2) regional stage, and 3) early stage/localized [34]. SEER statistics for the 10 cancer types included in the original scope of this review are presented in Table 4.

**Table 2:** HRQoL/ PRO instruments utilized in identified studies

Instrument	Details
**Generic PRO instruments**	
12-item anorexia/cachexia scale **(A/CS-12)**	• A/CS-12 is a subscale of the Functional Assessment of Anorexia–Cachexia Therapy (FAACT) [14]. • Scores range from –36 to 12 with higher scores indicating lower risk of cancer anorexia-cachexia syndrome and associated with better outcomes.
Apnea/Hypopnea Index **(AHI)**	• AHI measures the number of events of apnea/hypopnea per hour [15]. • Scores range from < 5 normal (no sleep apnea); 5–15 mild sleep apnea; 15–30 moderate sleep apnea; > 30 severe sleep apnea.
Eating assessment tool-10 **(EAT-10)**	• EAT-10 is a 10-item validated symptom-specific outcome instrument for dysphagia that addresses social, emotional, and functional implications of disability [16]. • Scores range from 0 to 40, with higher scores correlating with increasing severity of swallowing symptoms.
European Organization For Research And Treatment Of Cancer Core Quality of Life questionnaire **(EORTC QLQ-C30)**	• EORTC QLQ-C30 is designed to measure patients’ physical, psychological, and social functions [17]. • Scores range from 0 to 100 with a high functional/ global assessment score representing a high level of HRQoL or functioning whereas a high symptom score represents greater symptom burden.
European Quality of Life Five Dimension questionnaire **(EQ-5D-3L)**	• EQ-5D-3L consists of five dimensions (mobility, self-care, usual activities, pain/discomfort, and anxiety/depression), each with three levels (e.g., no problems, some problems, and extreme problems) [18]. • EQ-5D index score measures health status from − 1 (“worst imaginable health state”) to 1 (“best imaginable health state”). • EQ-5D health utility scores (HUS) scored three levels (no problems, some problems, severe problems) in reference to five dimensions: mobility, self-care, usual activities, pain or discomfort, and anxiety or depression. The combinations of answers were then reduced to a single health utility score that ranged typically from 0 (feeling as good as being dead) to 1 (having perfect heath).
Functional assessment of cancer therapy-General **(FACT-G)**	• Comprised of four subscales: physical well-being, social/family well-being), emotional well-being, and functional well-being [19]. • The mean score ranges from 0 to 108 with higher scores representing higher health related quality of life.
Hospital Anxiety and Depression Scale **(HADS)**	• Used to assess patient/client levels of Anxiety and Depression [20]. • Global scores 0–7 indicate absence of symptoms of anxiety or depression, scores 8–10 are inconclusive, and scores ≥ 11 indicate anxiety/depression.
MD Anderson Symptom Inventory **(MDASI)**	• The MDASI is a measure for assessing symptom burden [21]. • Patients rate the severity of 13 commonly experienced cancer-related symptoms and 6 items related to how much symptoms are interfering with activities of daily living. Scores for symptoms range from 0 to 10 with higher scores indicating greater symptom severity (0: symptom not present; 10: symptom as bad as you can imagine). Scores for symptom interference range from 0 to 10 with higher scores indicating greater symptom interference (0: does not interfere; 10: completely interferes).
National Comprehensive Cancer Network Distress Thermometer **(NCCN DT)**	• NCCN DT is a one item assessment that measures distress on an 11-point scale ranging from 0 (no distress) to 10 (extreme distress) [22].
**PERFORM**	• 12 item scale developed to assess fatigue in cancer patients [23]. • PERFORM scores range from 12–60, with higher scores indicating less fatigue.
Patient-Reported Outcomes Measurement Information System **(PROMIS)**	• Includes measures of: Anxiety (11 items); Depression (10 items); Fatigue (14 items); Pain Interference (10 items); Physical Function (15 items); Sleep Disturbance (10 items); Ability to Participate in Social Roles and Activities (version 2; 10 items; abbreviated as social function); and Cognitive Function (version 2; eight items) [24]. • PROMIS measures are standardized to a T-score metric (mean, 50; SD, 10) ranging from 20–80. • Higher scores reflect more of what is being assessed, either worse symptoms or higher levels of function.
Revised Psychosocial Screen for Cancer **(PSSCAN-R)**	• The PSSCAN‐R questionnaire is a validated 21‐item instrument to specifically screen for psychosocial distress among patients with cancer [25]. • The questionnaire contains questions pertaining to the patients' social support, their psychosocial needs through the Canadian Problem Checklist (CPC), and symptoms of anxiety and depression. • Scores less than 8 represent no symptoms of anxiety or depression, scores 8–10 represent subclinical symptoms, and a score ≥ 11 represents clinical symptoms.
Pittsburgh Sleep Quality Index **(PSQI)**	• PSQI is a self-rated questionnaire to assess sleep quality over a 1-month period [26]. • A global sleep score is generated through 19 individual items falling into 7 component categories: subjective sleep quality, sleep latency, sleep duration, habitual sleep efficiency, sleep disturbances, use of sleeping medication, and daytime dysfunction. Scores range from 0 to 21 with the higher total score (referred to as global score) indicating worse sleep quality.
National Surgical Adjuvant Breast and Bowel Project (NSABP) symptom checklist **(SCL-17)**	• Symptom burden questionnaire consisting of scores which are the average of 17 items scored on a 0 to 100 range with higher scores representing greater symptom burden [27].
Short-form survey-8 **(SF-8)**	• SF-8 is a self-reported assessment of HRQoL derived from the SF-36 instrument [28]. • Emotional problems item from the SF-8: patients are asked to rate their response to the following question “In the past month, how much have you been bothered by emotional problems, such as feeling anxious, depressed, or irritable?” Responses were scored on a 5-point scale (Not at All, Slightly, Moderately, Quite a Lot, Extremely). The presence of emotional problems was based on responses of moderate, quite a lot, or extreme problems.
Short-form survey-12 **(SF-12)**	• Generic quality of life questionnaires that measure physical, functional, emotional, and social wellbeing [29]. • Can be summarized into 2 indices: the Physical Component Summary (PCS) and the Mental Component Summary (MCS), describing patient physical and mental well-being, respectively. • For both PCS and MCS higher scores indicate better HRQoL, scores ≥ 50 suggest above average HRQoL compared to the general population, while scores < 50 suggest poor HRQoL. For MCS a score ≤ 42 has been recommended as a cut-off and may be indicative of clinical depression.
Short-form survey-36 **(SF-36)**	• The SF-36 Vitality subscale consists of four items and was designed to assess energy and fatigue [30]. • The SF-36 Vitality Scale is scored on a T-score metric (mean = 50, SD = 10, in US general population), with a higher score indicating more energy (range 0–100).
**Disease-specific PRO instruments**	
European Organization For Research And Treatment Of Cancer Core Quality of Life questionnaire – cervical cancer **(EORTC QLQ-CX24)**	• EORTC QLQ-CX24 is a cervical cancer specific module comprised of 24 items covering pain, bowel activities, urinary frequency, lymphodema, vaginal symptoms, hot flushes, body image, sexual activity, and sexual pain [31]. • Scores range from 0 to 100 with a high score representing a high response level. A high functional scale represents high/healthy functioning. Similarly, high global health scores represent a high HRQoL. • Conversely, a high score for a symptom scale represents higher symptom burden.
Functional assessment of cancer therapy-colorectal **(FACT-C)**	• FACT-C is a colorectal cancer module consisting of 36 items [32]. • Total score ranges from 0–136 with higher scores representing greater quality of life. • FACT-C TOI (trial outcome index) is derived from physical wellbeing, functional wellbeing, and colorectal cancer subscale scores. Scores range from 0–84, with higher scores indicating better HRQoL.
Functional assessment of cancer therapy-esophageal **(FACT-E)**	• FACT-E is a quality-of-life subscale of FACT-G, designed for patients with esophageal cancer [33]. FACT-E for esophageal cancer is FACT-G plus FACT-ECS. • FACT-E is comprised of five subscales: physical well-being, social/family well-being, emotional well-being, functional well-being, and esophageal cancer subscale. • Scores range from 0–176 with higher scores representing greater HRQoL.
Functional assessment of cancer therapy-esophageal cancer subscale **(FACT-ECS)**^**a**^	• FACT-ECS is a disease specific module/subscale comprised of 17 items addressing eating, swallowing, enjoyment of food, voice, dry mouth, appetite, cough, choking, and pain. Scores range from 0–68 with higher scores representing greater HRQoL.

*NSABP* National surgical adjuvant breast and bowel project

^a^FACT-ECS is a disease specific module/subscale, used in combination with FACT-G to generate FACT-E

**Table 3:** Summary of selected studies with results stratified by cancer stage

Reference	Study design	Staging classification	Instrument/PRO	Stage of disease (N)/ baseline mean score (SD) [SE]				*P value* (if applicable)	
**Distant Stage Cancers**									
*Lung cancer (n* = *6 studies)*									
Jensen (2017) [35]	Population based study	Disease stage (I-IV)	**PROMIS**^**a,b**^	**Stage I/II (308)**		**Stage III/IV (386)**			
			Pain interference^a^	54.2 [0.6]		55.8 [0.6]		NR	
			Fatigue^a^	54.6 [0.6]		58.2 [0.6]			
			Sleep disturbance^a^	51.1 [0.6]		52.6 [0.5]			
			Anxiety^a^	50.8 [0.7]		51.6 [0.6]			
			Depression^a^	50.1 [0.7]		51.4 [0.6]			
			Physical function^b^	40.2 [0.5]		37.9 [0.5]			
			Social function^b^	47.2 [0.6]		43.7 [0.6]			
			Cognitive function^b^	51.5 [0.7]		49.2 [0.7]			
Morrison (2017) [36]	Prospective	Disease stage (I-IV)	**SF-8 – emotional problems**^**c**^ (percentage of patients)	**Stage I (NR)**	**Stage II (NR)**	**Stage III (NR)**	**Stage IV (NR)**		
			Not at all (%)	47.4	8.9	23.2	20.5	*P* < 0.001	
			Slightly (%)	38	6.5	29.3	26.3		
			Moderately (%)	25	9.3	32.2	33.5		
			Quite a lot (%)	25.2	4.7	32.7	37.4		
			Extremely (%)	26.3	0	41.2	31.6		
Berry (2018) [37]	Longitudinal, single-arm study	Disease stage (I-IV)	**12-item anorexia/cachexia scale**^**d**^	**Stage I**	**Stage II**	**Stage III**	**Stage IV**		
			Low risk (score ≥ -4), number of patients (*n* = 44)	7	3	14	20	NR	
			Moderate to severe risk (score ≤ -4), number of patients (*n* = 46)	1	7	14	24		
			Median A/CS-12 score for moderate to severe risk patients	Median: -7.5 (-18 to -4)				*P* = 0.09	
Pierzynski (2018) [38]	NR	Disease stage (I-IV)	**SF-12**	**Stage I (621)**	**Stage II (228)**	**Stage III (979)**	**Stage IV (1768)**		
			SF-12-PCS^e^	43.9 (11.46)	43.68 (11.74)	41.16 (11.79)	37.74 (11.82)	• Stage I vs II *P* = 1.00 • Stage I vs III *P* < 0.001 • Stage I vs IV *P* < 0.001 *P* for trend *P* < 0.001	
			SF-12-MCS^e^	49.28 (10.39)	50.09 (10.37)	46.26 (11.21)	45.22 (11.52)	• Stage I vs II *P* = 0.988 • Stage I vs III *P* < 0.001 • Stage I vs IV *P* < 0.001 *P* for trend *P* < 0.001	
Leung (2019) [39]	NR	Without/ With Metastases (M0, M1)	**PSSCAN‐R**^**f**^	**M0 (2069) vs M1 (2212): Anxiety**		**M0 (2069) vs M1 (2212): Depression**		M0 vs M1: Anxiety	M0 vs M1: Depression
			Univariate Analysis OR (95%CI)	1.52 (1.29‐1.79)		1.24 (1.01‐1.51)		*P* < 0.001	*P* = 0.036
			Multivariate Analysis OR (95% CI)	1.46 (1.23‐ 1.73)		1.10 (0.95‐1.27)		*P* < 0.001	*P* = 0.196
Mendoza (2019) [40]	Retrospective analysis of data collected at initial intake	Early or Advanced stages		**Early stage (stage 0-IIB) (171)**		**Advanced stage (stage III-IVA) (289)**			
			**MDASI**^**g**^						
			Fatigue	2.2 (2.9)		3.9 (2.9)		*P* < 0.001	
			Disturbed Sleep	2.5 (2.9)		3.5 (2.9)		*P* < 0.001	
			Distress	2.4 (2.8)		3.5 (2.8)		*P* < 0.002	
			Pain	2.1 (3.1)		3.5 (3.4)		*P* < 0.001	
			Short breath	2.2 (2.7)		3.2 (3.2)		*P* < 0.001	
			Sadness	1.9 (2.7)		2.9 (3.1)		*P* < 0.002	
			Drowsiness	1.6 (2.6)		2.6 (2.9)		*P* < 0.001	
			Lack of appetite	1.3 (2.3)		2.1 (2.9)		*P* < 0.001	
			Dry mouth	1.2 (2.4)		1.9 (2.8)		*P* < 0.008	
			Difficulties remembering	1.2 (2.0)		1.1 (2.2)		*P* = 0.134	
			Numbness/tingle	1.1 (2.2)		1.1 (2.2)		*P* = 0.56	
			Nausea vomit	0.6 (1.9)		1.0 (2.2)		*P* = 0.08	
			Interfere work	2.3 (3.0)		4.4 (3.5)		*P* < 0.001	
			Interfere enjoy life	2.3 (3.0)		3.8 (3.4)		*P* < 0.001	
			Interfere gen activity	2.0 (2.8)		3.9 (3.3)		*P* < 0.001	
			Interfere mood	2.3 (2.8)		3.4 (3.1)		*P* < 0.001	
			Interfere walking	1.8 (2.7)		3.4 (3.4)		*P* < 0.001	
			Interfere relations others	1.2 (2.2)		2.2 (2.9)		*P* < 0.001	
			**Quality of life, single item scales**^**h**^						
			Social support	8.0 (3.1)		7.9 (3.1)		*P* = 0.69	
			Emotional well-being	7.1 (2.9)		6.5 (2.9)		*P* < 0.03	
			Overall quality of life	7.2 (2.8)		6.3 (2.8)		*P* < 0.001	
			Physical well-being	6.6 (2.6)		5.7 (2.9)		*P* < 0.002	
*Pancreatic cancer (n* = *2 studies)*									
Deng (2018) [41]	Prospective hospital-based cohort study	Disease stage (I-IV)		**Stage I (97)**	**Stage II (221)**	**Stage III (162)**	**Stage IV (533)**		
			**SF-12-MCS**^**e**^						
			Score ≥ 45.7, N (%)	36 (37.1%)	95 (43.0%)	45 (27.8%)	159 (29.8%)	*P* = 0.16	
			Score 32.7–45.7, N (%)	29 (29.9%)	59 (26.7%)	52 (32.1%)	187 (35.1%)		
			Score < 32.7, N (%)	32 (33.0%)	67 (30.3%)	65 (40.1%)	187 (35.1%)		
			OR unadjusted (95% CI)	1.00 (reference)	0.81 (0.52–1.27)	1.46 (0.92–2.34)	1.24 (0.83–1.86)		
			OR adjusted (95% CI)	1.00 (reference)	0.8 (0.5–1.27)	1.57 (0.96–2.55)	1.16 (0.75–1.78)		
			**SF-12 -PCS**^**e**^						
			Score ≥ 45.7, N	45 (46.4%)	103 (46.6%)	58 (35.8%)	140 (26.3%)	*P* < 0.001	
			Score 32.7–45.7, N	33 (34.0%)	66 (29.9%)	54 (33.3%)	184 (34.5%)		
			Score < 32.7, N	19 (19.6%)	52 (23.5%)	50 (30.9%)	209 (39.2%)		
			OR unadjusted (95% CI)	1.00 (reference)	1.07 (0.69–1.67)	1.64 (1.03–2.62)	2.47 (1.65–3.69)		
			OR adjusted (95% CI)	1.00 (reference)	1.08 (0.68–1.72)	1.8 (1.1–2.94)	2.32 (1.5–3.59)		
Ambai (2021) [42]	Retrospective study of available records with baseline MDASI	Disease stage (I-IV)	**MDASI**^**g**^	**Stage II/III (9)**		**Stage III/IV (50)**		NR	
			Mean MDASI score (maximum score 130)	47.3 (19.0)		51.8 (27.5)			
*Esophageal cancer (n* = *2 studies)*									
Doherty (2018) [43]	Cross-sectional survey study	Disease Stage (I-IV)		**Stage I** **(*N* = 1)**	**Stage II/III** **(*N* = 51)**		**Stage IV** **(*N* = 30)**		
			**EQ-5D-3L**^**i**^	0.71 (NA)	0.82 (0.13)		0.72 (0.18)	NR	
			**FACT-G**^**i**^	50.2 (NA)	82.3 (15)		72.5 (17)	NR	
			**FACT-E**^**i**^	91.2 (NA)	128.2 (27)		112.8 (25)	NR	
			**FACT-E-derived subscales**^**i**^						
			Physical wellbeing	-	23.4 (4.5)		20.6 (6.2)	NR	
			Emotional wellbeing	-	17 (4.3)		13.6 (6.3)		
			Social wellbeing	-	23.5 (5.5)		23.2 (4.6)		
			Functional wellbeing	-	18.4 (6.4)		15.8 (6)		
			Pain complex	-	3.2 (0.9)		2.8 (0.8)		
			Dysphagia complex	-	2.4 (1.1)		2.2 (1.2)		
			Nausea complex	-	3.4 (0.8)		3 (1)		
			Dyspnea complex	-	0.9 (0.4)		0.8 (0.4)		
			Loss of appetite complex	-	2 (1.4)		1.4 (1.3)		
			Fatigue complex	-	3.2 (0.8)		2.6 (0.9)		
			**FACT-ECS**^**i**^	41 (NA)	46 (13.8)		40.2 (12.2)	NR	
			**VAS**^**g**^	-	6.7 (2.3)		5.7 (2.4)	NR	
Kidane (2018) [44]	Prospective, non-randomized studies	T stage (1–4)		**T1** **(*N* = 10)**	**T2** **(*N* = 33)**	**T3** **(*N* = 79)**	**T4** **(*N* = 13)**		
			**FACT-E**^**i**^	81.7 (18)	78.1 (19)	75.3 (16.3)	78.4 (21)	*P* = 0.65	
			**FACT-ECS**^**i**^	58.7 (9.1)	45.6 (12.3)	42.3 (12.6)	44.5 (15.4)	*P* < 0.002	
*Stomach cancer (n* = *1 study)*									
Franck (2021) [45]	German multicenter cohort study	T stage (1–4)	**Cancer related symptoms questionnaire**	**Prevalence of Symptoms, n (%)**					
				**T1-2 (293)**		**T3-4 (215)**			
			Alarm symptoms (dysphagia, weight loss, bleeding, vomiting)	125 (44)		148 (68.8)		OR: 2.54 (1.77–3.66) *P* < 0.0001	
			Dyspepsia	187 (65.8)		151 (68)		OR: 1.1 (0.76–1.6) *P* = 0.64	
			Reflux	60 (21.1)		38 (17.1)		OR: 0.77 (0.49–1.21) *P* = 0.31	
		UICC stage (I-IV)		**Stage I-II (296)**		**Stage III-IV (219)**			
			Alarm Symptoms (dysphagia, weight loss, bleeding, vomiting)	127 (42.9)		152 (69.4)		OR: 3.02 (2.09–4.36) *P* < 0.0001	
			Dyspepsia	204 (68.9)		143 (65.3)		OR: 0.85 (0.59–1.23) *P* = 0.39	
			Reflux	64 (21.6)		34 (15.5)		OR: 0.67 (0.42–1.05) *P* = 0.09	
**Regional Stage Cancers**									
*Head and Neck cancer (n* = *4 studies)*									
Amin (2022) [46]	Prospective study	T-stage (0–4)	**EAT-10**^**j**^	**T0 (2)**	**T1 (21)**	**T2 (34)**	**T3 (9)**	*T1-T3: P* < 0.02 *T0 (2) and T4 (4) not included in statistical comparison due to small sample size*	
				0	2.38 (NR)	3.35 (NR)	10.3 (NR)		
				**T4 (4)**					
				1.75 (NR)					
Brauer (2021) [47]	Data from medical records were analyzed	Disease stage	**NCCN DT**^**k**^	**Stage 0 (9)**	**Early (103)**	**Locally Adv. (63)**	**Metastatic (28)**		
			Number of patients with DT score ≥ 4 (% patients)	5 (55.6%)	60 (58.3%)	41 (65.1%)	12 (42.9%)	NR	
			DT score	4.33 (3.74)	4.31 (3.27)	5.21 (3.33)	3.7 (3.58)		
Huppertz (2021) [48]	Prospective study	UICC (1–4)	**AHI**^**l**^	**Stage 1 (2)**	**Stage 2 (8)**	**Stage 3 (3)**	**Stage 4 (4)**		
				45.35 (0.35)	18.11 (20.6)	23.9 (21.42)	11.35 (9.80)	*P* = 0.2506	
Santoso (2021) [49]	Prospective study	Disease stage (I-IV)	**PSQI**^**m**^	**Stage I (134)**	**Stage II (103)**	**Stage III (90)**	**Stage IV (233)**		
			Good sleep, n (%)	84 (63)	59 (57)	45 (50)	126 (54)	*P* = 0.24	
			Poor sleep, n (%)	50 (37)	44 (43)	45 (50)	107 (46)		
*Colorectal cancer (n* = *6 studies)*									
Jensen (2017) [35]	Population based study	Disease stage (I-IV)	**PROMIS**^**a,b**^	**Stage I/II (442)**		**Stage III (292)**	**Stage IV (156)**		
			Pain interference^a^	52.1 [0.6]		54 [0.7]	56.5 [1]	NR	
			Fatigue^a^	50.8 [0.6]		53.9 [0.7]	56.5 [0.9]		
			Sleep disturbance^a^	50.2 [0.5]		51 [0.7]	50.9 [0.9]		
			Anxiety^a^	48.5 [0.6]		49.9 [0.7]	51 [1.1]		
			Depression^a^	47.6 [0.6]		48.9 [0.7]	49.8 [1]		
			Physical function^b^	46.5 [0.5]		43.4 [0.6]	41.8 [0.9]		
			Social function^b^	51.2 [0.6]		48 [0.7]	45.4 [1]		
			Cognitive function^b^	52.9 [0.6]		49.7 [0.7]	49.1 [1.1]		
Reyes (2017) [50]	NR	Disease stage (I-IV)	**SF-12**	**Stage I (146)**	**Stage II (286)**	**Stage III (568)**	**Stage IV (372)**		
			SF-12-MCS^e^	50.1 (10.5)	49.5 (10.5)	48.9 (10.2)	46.0 (10.9)	*P* = 4.88 × 10^−6^	
			SF-12-PCS^e^	46.9 (9.6)	45.7 (10.6)	46.9 (10.1)	40.8 (11.8)	*P* = 5.79 × 10^−30^	
Belachew (2020) [51]	Retrospective analysis of CRC cases identified from the MD Anderson Cancer Patient and Survivors Cohort	Disease stage (I-IV)	**SF-12**	**Stage I (W: 43; H: 23; B: 32)**	**Stage II (W: 73; H: 74; B: 54)**	**Stage III (W: 143; H: 134; B: 100)**	**Stage IV (W: 108; H: 83; B: 86)**		
			**SF-12-MCS**^**e**^						
			White	49.9 (10.9)	49.6 (10.2)	48.9 (9.8)	47.5 (10.7)	*P* = 0.45	
			Hispanic	50.2 (9.3)	47.9 (10.9)	47.4 (11.1)	48.0 (11.0)	*P* = 0.73	
			Black	48.0 (12.4)	48.7 (10.6)	49.3 (10.3)	46.3 (12.2)	*P* = 0.33	
			**SF-12-PCS**^**e**^						
			White	46.7 (11.2)	45.5 (10.1)	44.6 (10.1)	40.3 (11.0)	P < 0.05	
			Hispanic	47.1 (9.7)	43.7 (11.1)	43.6 (10.2)	37.2 (11.4)	P < 0.05	
			Black	44.9 (11.3)	42.1 (11.6)	39.6 (12.0)	37.3 (11.7)	P < 0.05	
Ganz (2022) [52]	HRQoL substudy	Disease stage (I-IV)		**Stage II (815)**		**Stage III (557)**			
			**FACT-C TOI**^**i**^	66.1 (NR)		64 (NR)		*P* < 0.004	
			**SCL-17**^**n**^	7.2 (NR)		8.5 (NR)		*P* < 0.001	
			**SF-36-vitality**^**o**^	53.5 (NR)		52.8 (NR)		*P* = 0.179	
Ruiz-Casado (2022) [53]	Cross-sectional	Disease stage (I-IV)	**PERFORM questionnaire**	**Stage II/III (111)**		**Stage IV (77)**			
			Assessment of fatigue^p^	51.4 (NR)		50.5 (NR)		NR	
Varela‐Moreno (2022) [54]	Prospective, observational, multicenter cohort study	TNM staging	**HADS**^**q**^ **(Number of Patients)**	**T stage (T0-T2: 688; T3-T4: 1598)**	**N stage (N0: 1415; N1-N2: 864)**	**Without/With Metastases (M0: 1629; M1: 181)**			
			HADS-D Normal (< 8)	T0/T1/T2: 545 T3/T4: 1206	N0: 1102 N1: 647	Without: 1261 With: 125		T stage: *P* = 0.059 N stage: *P* = 0.112 M stage: *P* < 0.015	
			HADS-D Positive (> 11)	T0/T1/T2: 143 T3/T4: 392	N0: 313 N1: 217	Without: 368 With: 56			
**Localized Cancers**									
*Anal cancer (n* = *1 study)*									
Sauter (2016) [55]	Retrospective analysis of all patients referred to Triemli-Hospital for treatment of anal carcinoma	T (1–4)	**Presenting symptoms**	**T1 (*N* = 8)**	**T2 (*N* = 32)**	**T3 (*N* = 25)**	**T4 (*N* = 21)**	χ2	
			Total symptoms, n	2.12	2.75	3.52	4.43	*P* < 0.01	
			Blood in stool, %	75	77	84	76	*P* = 0.78	
			Painful defecation, %	25	26	52	48	*P* = 0.26	
			Anal pain, %	25	29	24	33	*P* = 0.83	
			Perianal pain, %	0	10	32	48	*P* < 0.01	
			Outlet obstruction, %	0	3	12	10	*P* = 0.20	
			Incontinence, %	0	6	16	19	*P* = 0.07	
			Pencil stool, %	0	6	4	10	*P* = 0.45	
			Diarrhea, %	0	10	16	10	*P* = 0.50	
			Irregular stool, %	0	6	8	10	*P* = 0.40	
			Constipation, %	0	0	8	14	*P* < 0.02	
			Foreign body sensation, %	0	2	20	29	*P* = 0.28	
			Pruritus, %	37	29	16	5	*P* < 0.01	
			Tumor on self-palpation, %	25	26	24	29	*P* = 0.81	
			Abdominal pain, %	0	0	4	14	*P* < 0.02	
			Mechanical ileus, %	0	0	0	5	*P* = 0.16	
			Vaginal stool, %	0	0	0	5	*P* = 0.16	
			Inguinal lymph nodes on self-palpation, %	0	3	0	5	*P* = 0.64	
			Weight loss, %	25	20	30	60	*P* < 0.01	
			Anemia, %	0	0	4	5	*P* = 0.22	
			Asymptomatic, %	12	0	0	0	*P* = 0.63	
*Cervical cancer (n* = *3 studies)*									
Ferrandina (2012) [56]	Prospective, longitudinal study	FIGO staging		**Early stage (FIGO stage IA (28); IB (77))**		**Locally advanced (FIGO stage IB-IA bulky (16); IIIB (86); IIIA/IV (20))**			
			**HADS**^**q**^						
			Anxiety score	7.8 [0.4]		7.7 [0.3]		*P* = 0.835	
			Depression score	3.3 [0.3]		3.8 [0.3]		*P* = 0.244	
			**EORTC QLQ-CX24**^**i,g**^						
			Body image^i^	13.6 [2.1]		13.1 [1.7]		*P* = 0.83	
			Lymohedema^g^	5.7 [2.1]		2.9 [0.8]		*P* = 0.061	
			Menopausal symptoms^g^	12.7 [2.3]		11.4 [1.9]		*P* = 0.671	
			Neuropathy^g^	11.6 [1.7]		11.4 [1.7]		*P* = 0.633	
			Sexual Activity^i^	17.1 [2.4]		9.1 [1.6]		*P* < 0.004	
			Sexual Enjoyment^i^	52.1 [4.5]		22.9 [4.6]		*P* < 0.006	
			Sexual Functioning^i^	6.6 [1.7]		8.4 [1.9]		*P* = 0.533	
			Sexual Worry^g^	16.9 [2.9]		16.6 [2.3]		*P* = 0.954	
			Symptom Experience^g^	11.1 [1.2]		12.6 [0.9]		*P* = 0.315	
			**EORTC-QLQ-C30**^**i**^						
			Global/overall	76.1 [2.2]		72.9 [1.7]		*P* = 0.264	
Mantegna (2013) [57]	Prospective, longitudinal study	FIGO staging	**HADS**^**q**^	**Early stage** **FIGO stage IB-IIA (tumor < 4 cm) (105)**		**Locally advanced** **FIGO stage IB-IIA (tumor > 4 m) or IIB-IVA (122)**			
			Anxiety level ≥ 11, %	25.7%		22.2%		Anxiety level: *P* = 0.443 Depression level: *P* = 0.669	
Jensen (2017) [35]	Population based study	Disease stage (I-IV)	**PROMIS**^**a,b**^	**Stage I (80)**	**Stage II/II/IV (61)**				
			Pain interference^a^	51.1 [1.4]	56.1 [1.7]			NR	
			Fatigue^a^	51.8 [1.6]	56.6 [1.8]				
			Sleep disturbance^a^	52.5 [1.2]	54.2 [1.4]				
			Anxiety^a^	51.5 [1.6]	54.9 [1.7]				
			Depression^a^	50.0 [1.6]	53.6 [1.8]				
			Physical function^b^	48.4 [1.3]	41.2 [1.2]				
			Social function^b^	52.6 [1.6]	46.9 [1.5]				
			Cognitive function^b^	51.7 [1.5]	47.9 [1.6]				

*Abbreviations:* *A/CS-12* 12-item anorexia/cachexia scale, *AHI* Apnea/Hypopnea Index, *EAT-10* Eating assessment tool-10, *EORTC QLQ-C30* EORTC core quality of life questionnaire, *EORTC QLQ-CX24* EORTC Quality-of-Life questionnaire cervical cancer module, *EORTC* European Organisation for Research and Treatment of Cancer, *EQ-5D-3L* European Quality of Life 5 Dimensions 3 Level Version, *FACT* Functional assessment of cancer therapy, *FACT-C* FACT-colorectal cancer, *FACT-E* FACT-esophageal cancer, *FACT-ECS* FACT-esophageal cancer subscale, *FACT-G* FACT-General, *HADS* Hospital Anxiety and Depression Scale, *MCS* mental composite summary, *MDASI* MD Anderson Symptom Inventory, *NCCN DT* National Comprehensive Cancer Network Distress Thermometer, *NR* not reported, *OR* odds ratio, *PCS* physical composite summary, *PRO* patient reported outcome, *PROMIS* Patient-Reported Outcomes Measurement Information System, *PSQI* Pittsburgh Sleep Quality Index, *PSSCAN-R* Psychosocial Screen for Cancer-Revised, *SCL-17* NSABP Symptom Checklist, *SD* standard deviation, *SE* standard error, *SF-8* Short Form 8 Health Survey, *SF-12* 12-Item Short Form Survey, *SF-36* Short Form-36, *TNM* tumor, node, metastasis, *TOI* Trial outcome index, *VAS* visual analogue scale

*P* < 0.05 considered statistically significant

^a^Higher score indicates higher symptom severity

^b^Higher score indicates better function

^c^Scores are grouped by prevalence of emotional problems

^d^Higher scores indicate lower risk

^e^Higher scores reflect more of what is being assessed, either worse symptoms or higher levels of function

^f^Scores ≥ 11 indicate clinical symptoms

^g^Higher scores indicate worse HRQoL

^h^Lower scores indicate worse HRQoL

^i^Higher scores indicate better HRQoL

^j^Higher scores indicate greater swallowing problems

^k^Higher scores indicate more distress

^l^Higher scores indicate more severe apnea

^m^Results stratified into good versus poor sleep

^n^Higher scores indicate greater burden

^o^Higher score indicate more vitality

^p^Higher scores indicate less fatigue

^q^Scores ≥ 11 indicate clinical symptoms

**Table 4:** Cancer statistics in the United States

Cancer Type	Prevalence of Cases in 2020	Incidence Rate (per 100,000) in 2023	Estimated Deaths in 2023	Stage at diagnosis	5-Year Relative Survival Overall	5-Year survival by stage at diagnosis	Lifetime risk
**Distant Stage**							
**Ovarian**	236,511	10.3	13,270	Localized: 18% Regional: 20% Distant: 55% Unknown: 6%	50.8%	Localized: 92.4% Regional: 72.9% Distant: 31.5% Unknown: 36.4%	1.1%
**Lung**	603,989	50.0	127,070	Localized: 21% Regional: 21% Distant: 53% Unknown: 5%	25.4%	Localized: 62.8% Regional: 34.8% Distant: 8.2% Unknown: 15.1%	6.1%
**Pancreatic**	95,389	13.3	50,550	Localized: 13% Regional: 29% Distant: 51% Unknown: 7%	12.5%	Localized: 44.3% Regional: 16.2% Distant: 3.2% Unknown: 10.5%	1.7%
**Esophageal**	50,379	4.2	16,120	Localized: 18% Regional: 33% Distant: 38% Unknown: 11%	21.7%	Localized: 48.8% Regional: 27.7% Distant: 5.6% Unknown: 16.5%	0.5%
**Stomach**	127,211	6.9	11,130	Localized: 29% Regional: 25% Distant: 36% Unknown: 10%	35.7%	Localized: 74.7% Regional: 34.6% Distant: 6.6% Unknown: 31.1%	0.8%
**Regional Stage**							
**Head and Neck**	424,284	11.4	11,580	Localized: 27% Regional: 51% Distant: 15% Unknown: 7%	68.5%	Localized: 86.6% Regional: 69.1% Distant: 39.3% Unknown: 61.2%	1.2%
**Colorectal**	1,388,422	36.6	52,550	Localized: 35% Regional: 36% Distant: 23% Unknown: 6%	65%	Localized: 90.9% Regional: 73.4% Distant: 15.6% Unknown: 48.6%	4.1%
**Localized Stage**							
**Anal**	79,091	1.9	1,870	Localized: 43% Regional: 35% Distant: 13% Unknown: 9%	70.4%	Localized: 83.7% Regional: 67.5% Distant: 36.2% Unknown: 66.9%	0.2%
**Cervical**	296,981	7.7	4,310	Localized: 43% Regional: 36% Distant: 15% Unknown: 7%	67.2%	Localized: 91.2% Regional: 59.8% Distant: 18.9% Unknown: 61.8%	0.7%
**Liver and bile duct**	105,765	9.3	29,380	Localized: 42% Regional: 26% Distant: 19% Unknown: 12%	21.6%	Localized: 37.3% Regional: 14.3% Distant: 3.5% Unknown: 10.5%	1.1%

Source: SEER explorer https://seer.cancer.gov/statistics-network/explorer/

Incidence rates are age-adjusted and based on 2016–2020 cases. Incidence rates are described per 100,000 men and women for all cancers except cervical and ovarian cancer which are described per 100,000 women. Head and neck cancer statistics are for cancers of oral cavity and pharynx and may not be fully inclusive of all head and neck cancers described in this review

Disease staging varied throughout the 26 included studies and is reported as described in each study. Disease staging may have been described descriptively (ex. early-stage vs advanced stage) or according to a staging system such as the TNM Classification of Malignant Tumors (TNM) developed by the Union for International Cancer Control (UICC). The TNM is used for describing cancer based on: 1) tumor size and tissue location (T0 indicating no evidence of a tumor and T1-T4 describing the progressive size and invasiveness), 2) spread to lymph nodes (N0 indicating no regional nodal spread and N1-N3 indicating progressively distal nodal spread), and 3) presence of metastases (M0 indicating no metastases and M1 indicating presence of metastases) [58]. The combination of these 3 factors from the TNM system can then be used for simplified cancer staging (Stages I, II, III, and IV) [58]. While categorization as early or advanced disease based on staging varies between cancer types, generally Stage I indicates localized cancer (T1-T2, N0, M0), stage II indicates early-stage locally advanced cancer (T2-T4, N0, M0), stage III indicates late-stage locally advanced cancer (T1-T4, N1-N3, M0), and stage IV indicates metastatic cancer (T1-T4, N1-N3, M1) [58].

### Cancers predominantly diagnosed at distant stage

#### Lung cancer

The primary stage of diagnosis for lung cancer is at a distant stage, accounting for 53% of diagnoses [34]. For lung cancer, HRQoL and symptom burden by disease stage was reported using PRO instruments including Patient-Reported Outcomes Measurement Information System (PROMIS) [35], Short-Form Survey-8 (SF-8) [36], 12-item anorexia/cachexia scale (A/CS-12) [37], Short-Form Survey-12 (SF-12) [38], Revised Psychosocial Screen for Cancer (PSSCAN-R) [39], MD Anderson Symptom Inventory (MDASI) [40], and quality of life (QoL) single item scales [40]. Overall, findings suggested that both physical and mental HRQoL were impaired in advanced stages compared with early-stage disease.

Patients with stage III or IV disease reported significantly poorer physical and mental HRQoL versus patients with stage I disease [38]. Physical HRQoL scores were 41.16 and 37.74 in patients with stage III or IV disease and 43.9 in patients with stage I disease (SF-12, *P* for trend < 0.001). Mental HRQoL scores were 46.26 and 45.22 in patients with stage III or IV disease and 49.28 in patients with stage I disease (SF-12, p for trend < 0.001). Additionally, between patients with advanced versus early stage disease, poorer HRQoL measured using single-item QoL scales was reported for emotional well-being (6.5 vs 7.1, *P* < 0.03), physical well-being (5.7 vs 6.6, *P* < 0.002), and overall QoL (6.3 vs 7.2, *P* < 0.001) [40].

A correlation between advanced disease stage and poorer mental health was also reported in studies using other PRO instruments. Advanced disease stage was significantly associated with an increased prevalence of emotional problems (SF-8, *P* < 0.001) [36]. Additionally, anxiety was more prevalent in patients with metastases versus those without metastases (PSSCAN-R, Odds Ratio (OR): 1.46, *P* < 0.001), although this association was not found for depression (Odds Ratio (OR): 1.10, *P* = 0.196) [39].

Greater symptom prevalence and impact were also associated with patients with advanced disease. Patients with stage III/IV disease reported worse fatigue versus patients with stage I/II disease (PROMIS, 54.6 vs 58.2) based on a clinically meaningful difference of 3 points (as defined by the study authors for the PROMIS instrument) [35]. Social function was also worse in patients with stage III/IV disease (47.2 vs 43.7), indicating that the higher symptom burden reported by patients with advanced disease also has a broader impact on patient functioning [35]. These results are supported by a second study that reported a greater prevalence of both physical and emotional symptoms (measured using the MDASI) in patients with advanced disease compared with patients with early-stage disease [40]. Symptoms significantly associated with advanced disease included sleep problems (3.5 vs 2.5, *P* < 0.001), drowsiness (2.6 vs 1.6, *P* < 0.001), fatigue (3.9 vs 2.2, *P* < 0.001), sadness (2.9 vs 1.9, *P* < 0.002), pain (3.5 vs 2.1, *P* < 0.001), shortness of breath (3.2 vs 2.2, *P* < 0.001), lack of appetite (2.1 vs 1.3, *P* < 0.001), and dry mouth (1.9 vs 1.2, *P* < 0.008). Advanced disease was also associated with increased symptom interference for the domains of work (4.4 vs 2.3, *P* < 0.001), enjoying life (3.8 vs 2.3, *P* < 0.001), general activity (3.9 vs 2.0, *P* < 0.001), mood (3.4 vs 2.3, *P* < 0.001), walking (3.4 vs 1.8, *P* < 0.001), and relationships with others (2.2 vs 1.2,* P* < 0.001). Risk for anorexia/cachexia was not significantly associated with disease stage (A/CS-12, *P* = 0.09) [37].

#### Pancreatic cancer

The primary stage of diagnosis for pancreatic cancer is at distant stage, accounting for 51% of diagnoses [34]. HRQoL and symptom outcomes in patients with pancreatic cancer were evaluated using the SF-12 [41] and MDASI [42]. Worse physical HRQoL was associated with advanced tumor stage (I-IV) (SF-12, *P* for trend < 0.001), although this association was not significant for mental HRQoL (SF-12, *P* for trend 0.16) [41]. Additionally, patients in stage III/IV had higher symptom scores compared with patients in stage II/III (MDASI, 51.8 vs 47.3), indicating worse symptom severity, although no statistical tests or *P* values were reported [42].

#### Esophageal cancer

The primary stage of diagnosis for esophageal cancer is at distant stage, accounting for 38% of diagnoses [34]. For esophageal cancer, results stratified by disease stage were reported for the instruments Functional Assessment of Cancer Therapy-General (FACT-G) [43], FACT-Esophageal (FACT-E) [43, 44], FACT-Esophageal Cancer Subscale (FACT-ECS) [43, 44], and European Quality of Life Five Dimension questionnaire (EQ-5D-3L) [43].

Better HRQoL was reported in patients with stage II/III disease versus patients in stage IV [43]. Patients with stage II/ III disease reported a mean (SD) EQ-5D baseline utility score of 0.82 (0.13) compared with a score of 0.72 (0.18) in patients with stage IV or recurrent disease. Given a minimally important difference in EQ-5D Health Utility Score (HUS) of 0.07, this indicates that patients with stage IV or recurrent disease have clinically meaningful impaired HRQoL compared with patients with early-stage disease [43]. Poorer HRQoL with advanced disease stage was also reported using disease specific instruments. Patients in stage IV showed directionally poorer scores versus patients in stage II/III for symptoms associated with esophageal cancer (FACT-ECS, 40.2 vs 46.0) and HRQoL subscales such as emotional well-being (FACT-E, 13.6 vs 17.0). However, p values were not reported for these comparisons [43]. A statistically significant trend between higher T-stage and worse HRQoL was reported between patients with T4 disease versus T1 (FACT-ECS, 44.5 vs 58.7, *P* < 0.002), however this trend was not significant for all instruments (FACT-E, *P* = 0.65) [44].

#### Stomach cancer

The primary stage of diagnosis for stomach cancer is at distant stage, accounting for 36% of diagnoses [34]. A significantly greater prevalence of reported cancer symptoms was associated with advanced disease stage, with results stratified by both T stage (1–4) and UICC stage (I-IV) [45]. A higher prevalence of alarm symptoms (dysphagia, weight loss, bleeding, vomiting) was reported by patients with T-stage 3/4 versus T-stage 1/2 (OR: 2.54, *P* < 0.0001), and for patients with UICC stage III/IV versus UICC stage I/II (OR: 3.02, *P* < 0.0001).

### Cancers predominately diagnosed at regional stage

#### Head and neck cancer

The primary stage of diagnosis for head and neck cancer is at regional stage, accounting for 51% of diagnoses [34]. For head and neck cancer, results stratified by disease stage were reported for the PRO instruments National Comprehensive Cancer Network Distress Thermometer (NCCN DT) [47], Pittsburgh Sleep Quality Index (PSQI) [49], Apnea/Hypopnea Index (AHI) [48], and Eating Assessment Tool-10 (EAT-10) [46]. There was a significant correlation between advanced disease (higher T stage) and problems with swallowing (i.e., increased severity of swallowing impairment; EAT-10, *P* < 0.02) [46]. No statistically significant differences based on disease stage were reported for distress (NCCN DT) [47], sleep quality [49], or apnea and hypoxia [48].

#### Colorectal cancer

The primary stage of diagnosis for colorectal cancer is at regional stage, accounting for 36% of diagnoses [34]. In colorectal cancer, HRQoL and symptom burden by disease stage was reported using a range of PRO assessments including SF-12 [50, 51], PROMIS [35], FACT-Colorectal (FACT-C) and NSABP Symptom Checklist (SCL-17) [52], PERFORM fatigue questionnaire [53], and Hospital Anxiety and Depression Scale (HADS) [54]. Across all PRO assessments, advanced stage disease was generally associated with poorer HRQoL and increased symptomology and burden.

Significantly poorer physical and mental HRQoL was reported for patients with late-stage versus early-stage disease [50]. Comparing between patients in stage IV versus stage I, advanced disease was associated with significantly lower scores (poorer HRQoL) for both physical HRQoL (SF-12 PCS, 40.8 vs 46.9, *P* < 0.001) and mental HRQoL (SF-12, 46.0 vs 50.1, *P* < 0.001). Additionally, another study also evaluated ethnicity (white, black, or Hispanic) as a factor in HRQoL outcomes [51]. While advanced disease stage in all ethnicities was significantly associated with poorer physical HRQoL (SF-12 PCS, *P* < 0.05, for all), worse mental HRQoL with advanced disease was not observed, regardless of ethnicity. In comparing HRQoL in patients with stage II vs stage III cancer, significantly poorer HRQoL was reported in patients with stage III cancer (FACT-C TOI, 66.1 vs 64.0, *P* < 0.004) [52].

An increase in symptoms and the impact of symptoms on functioning were also associated with advanced stage disease. Clinically meaningful differences (defined by the study authors as a difference of 3 points for the PROMIS instrument) were reported in patients with stage IV and stage III cancer versus stage I/II across a range of functions and symptoms. Poorer functioning was reported in patients with stage III and IV disease versus patients with stage I/II for physical (41.8 and 43.4 vs 46.5), social (45.4 and 48.0 vs 51.2), and cognitive function (49.1 and 49.7 vs 52.9) [35]. Symptoms of pain (56.5 vs 52.1, stage IV vs stage I/II) and fatigue (56.5 vs 50.8, stage IV vs stage I/II) were also clinically worse in patients with advanced disease [35]. However, other studies reported no significant difference in fatigue between early and advanced-stage disease using other PRO instruments (SF-36 vitality subscale [52] and PERFORM 12-item scale [53]). Symptom impact was also significantly associated with advanced disease stage. Patients with stage III reported greater symptom impact (pain, vision and hearing problems, and GI problems) compared with patients with stage II cancer (SCL-17, 8.5 vs 7.2, *P* < 0.001) [52]. Additionally, depression was significantly more prevalent in patients with metastatic disease versus those without (HADS, 31% vs 23%, *P* < 0.015), although this association was not statistically significant when comparing patients based on T stage or N stage [54].

### Cancers predominately diagnosed at localized stage

#### Anal cancer

The primary stage of diagnosis for anal cancer is at localized stage, accounting for 43% of diagnoses [34]. For anal cancer, symptom burden was described stratified by T-stage (T 1–4) [55]. Overall, the most common symptoms reported by patients with anal cancer were anal bleeding (78%), anal/perianal pain (29% and 24%, respectively), weight loss (31%), tumor on self-examination (26%), and foreign body sensation (22%). Patients with locally advanced cancer (T3/T4) reported significantly greater prevalence of constipation and abdominal pain (*P* < 0.02), and perianal pain and weight loss (*P* < 0.01). Meanwhile, pruritus was significantly more frequent in patients with early T stages (*P* < 0.01). Patients with more advanced disease reported significantly more symptoms than those with less advanced tumors (average total number of symptoms for T1 vs T4, 2.1 vs 4.4, *P* < 0.01) indicating overall poorer HRQoL.

#### Cervical cancer

The primary stage of diagnosis for cervical cancer is at localized stage, accounting for 43% of diagnoses [34]. For cervical cancer, results stratified by disease stage were reported for the PRO instruments European Organization For Research And Treatment Of Cancer Core Quality of Life questionnaire (EORTC QLQ-C30) and EORTC cervical cancer questionnaire (EORTC QLQ-CX24) [56], PROMIS [35], and HADS [56, 57]. In terms of global HRQoL (measured using EORTC QLQ-C30), there was no statistically significant association between cancer stage, early vs locally advanced, and global score (72.9 vs 76.1, *P* = 0.264). Symptom burden was assessed through the PRO instruments, PROMIS, and HADS, and the disease-specific EORTC QLQ-CX24 instrument. Patients with locally advanced cancer reported significant impairments versus patients with early stage for sexual activity (EORTC QLQ-CX24, 9.1 vs 17.1, *P* < 0.004) and sexual enjoyment (EORTC QLQ-CX24, 22.9 vs 52.1, *P* < 0.006) [56]. Poorer mental and physical HRQoL was reported between patients with stage I cancer versus stage II/III/IV cancer for the PROMIS domains of pain interference (51.1 vs 56.1), fatigue (51.8 vs 56.6), anxiety (51.5 vs 54.9), depression (50.0 vs 53.6), physical function (48.4 vs 41.2), social function (52.6 vs 46.9), and cognitive function (51.7 vs 47.9) [35]. Depression and anxiety were evaluated based on FIGO staging in two studies [56, 57]. No significant differences were reported for either anxiety or depression, although one study noted a directional trend of more patients with locally advanced disease reporting anxiety compared with patients with early-stage disease (HADS, 63% vs 53%) [56].

## Discussion

In this narrative literature review of patient reported outcomes assessing symptom impact and health-related quality of life across 10 different cancer types, a general trend was observed for worse PRO results in patients with cancer diagnosed at an advanced stage of disease versus patients diagnosed at an earlier stage. Advanced disease stage was associated with greater prevalence of symptoms and increased symptom impact including general physical impairments such as pain, fatigue, and interference with functioning, as well as disease/region-specific symptom burden. Poorer HRQoL was also associated with advanced disease with commonly reported symptoms including anxiety and depression.

HRQoL, measured using generic PRO instruments, was worse in patients with advanced stage disease compared with patients with early-stage disease across cancer types. A range of generic instruments were used to measure HRQoL including the SF-12, reported in 4 studies [36, 39, 46, 48]. Clinically meaningful differences in HRQoL and symptoms were also reported for other PRO instruments such as PROMIS, with patients with advanced stage disease reporting worse HRQoL and more symptoms than patients with early stage disease [35]. Increased prevalence and/or interference of pain was correlated with advanced disease stage for colorectal cancer [35], lung cancer [35, 40], cervical cancer [35], and anal cancer [55]. This included both general pain [35, 40], and cancer type/site-specific pain such as perianal and abdominal pain in anal cancer [55]. Other general symptoms correlated with advanced disease included fatigue and sleep disturbances in lung, colorectal, and cervical cancers [35, 40], impairments in physical and cognitive function in colorectal and cervical cancer [35], nausea, lack of appetite, or anorexia and cachexia for lung cancer [37] and anal cancer [55].

The results of this review also highlight the increased burden of disease-specific symptoms in patients with advanced stage disease. Results from cancer-type specific PRO instruments were reported for the cancer types of colorectal (FACT-C TOI), cervical (EORTC QLQ-CX24), and esophageal (FACT-ECS) cancers. For both colorectal and esophageal cancer, a statistically significant association was reported between advanced cancer stage and worse HRQoL scores, as measured by the relevant disease-specific PRO instrument [44, 52]. For cervical cancer, advanced cancer stage was associated with statistically significant impairments in sexual activity and enjoyment [56]. In addition, disease-specific symptoms that correlated with advanced stage disease were seen in stomach cancer for alarm symptoms (dysphagia, weight loss, bleeding, vomiting) [45], and in head and neck cancer with trouble swallowing [46]. Together, these findings highlight the importance of using disease-specific PRO instruments to assess HRQoL and support the previously published observation that disease specific instruments are likely more sensitive to detect differences both between therapies [59, 60], but also, in the case of this review, when comparing between patients in different disease stages [59, 60].

Increased symptom burden can also impact a patient’s functional status, such as physical, emotional, or social functioning. Findings from this review show that advanced disease was associated with increased symptom interference in multiple areas including ability to work, walk, and general activity in lung cancer [40], and greater overall symptom burden in colorectal cancer [52] and anal cancer [55]. Increased prevalence or severity of emotional problems with more advanced disease was reported for multiple cancer types. Increased prevalence of emotional problems was correlated with advanced cancer stage for colorectal cancer [35, 50, 54], lung cancer [36, 38–40], and cervical cancer [35]. Statistically significant trends for worse mental HRQoL with advanced disease stage were also reported for colorectal cancer [46] and lung cancer [36]. Additionally, there was a statistically significant association between metastatic disease in colorectal cancer and prevalence of depression, although results were non-significant when stratified by T-stage or N-stage [52]. However, in cervical cancer, a statistically significant association was not found between advanced disease stage and prevalence of anxiety or depression [54, 55].

Across the 10 cancer types assessed in this review, 8 cancer types reported PRO results stratified by disease stage, while no studies were identified for the cancer types of liver and bile duct, or ovarian cancer. The number of studies found with results stratified by disease stage varied between cancer types and this is likely impacted by multiple variables including cancer prevalence and incidence, screening availability, distribution of cancer stage at diagnosis, treatment options, and efficacy and survival rates. The greatest number of studies found were for lung and colorectal cancer, returning 6 results each. Perhaps unsurprisingly, these cancers are the most prevalent of the 10 cancers included in the scope of this review: 1,388,422 for colorectal cancer, and 603,989 for lung cancer (Table 4) [34]. In contrast, no studies with results stratified by disease stage were found for the cancer types liver and bile duct, and ovarian, despite relatively high U.S. prevalence rates, 105,765 and 236,511, respectively (Table 4) [34]. The lack of results for these two cancers may be due to the lack of screening paradigms available for these cancers, thus resulting in the majority of cases being detected in later stages. Among the 10 cancers included in the scope of this narrative literature review, the stage at which each cancer type is primarily diagnosed varies. While stage distribution at diagnosis for colorectal cancer is more evenly distributed between early/localized stage (35% of cases), regional stage (36% of cases), and late/distant stage (23% of cases), other cancers are more highly skewed towards diagnosis at the advanced (distant) stage, including lung (53% of cases), pancreatic (51% of cases), esophageal (38% of cases), stomach (36% of cases), and ovarian (55% of cases) (Table 4) [34]. In general, 5-year survival rates are greater for those cancers that are more often diagnosed in earlier stages, although exceptions apply (e.g., liver and bile duct) [2]. Overall, data support the importance of early diagnosis and treatment to improve survival rates and reduce the negative impact of late diagnosis on patient symptom burden and HRQoL.

A few key limitations are present in this narrative literature review. First, while database searches were conducted in a systematic manner, this work was not intended to be a systematic review. Therefore, the studies selected are considered to be of most relevance to the question being addressed but may not include all relevant references. While the primary objective of this narrative literature review was to identify and collate published literature on patient burden at different stages of disease progression for the ten selected cancers, the secondary objective was to evaluate HRQoL based on cancer type and stage, within and between different cancer types. However, selected literature was heterogenous in terms of patient populations and study design. This review included both prospective and retrospective studies, the latter of which carries additional limitations including the potential for bias due to missing or misreported data. Also, while this review was focused on identifying patients with PRO assessments at the time of diagnosis and prior to treatment, the nature of retrospective claims analyses means that it is sometimes difficult to determine if patients may have previously received treatment. Additionally, studies may not have been powered for PRO endpoints. Statistical comparisons were not reported in all studies and few studies reported minimally important differences. Taken together, these factors limited the ability to draw strong conclusions.

## Conclusions

The findings of this narrative literature review support the search for improvements in cancer screening and earlier detection and treatment. Studies with results stratified by disease stage were limited, likely due to some cancers primarily being detected at advanced stages. Although the HRQoL data lacked consistent stratification by cancer stage, advanced stage cancer at diagnosis and prior to treatment was generally associated with worse HRQoL. This observation was expected due to stage or spread of disease likely playing a significant role in symptom impact burden. Overall, this supports the importance of detecting and treating cancer at earlier stages when patients may be asymptomatic or have lower symptom burden to minimize the increased negative impact on HRQoL and functional status observed in cancers diagnosed in advanced stage.

## Data Availability

No datasets were generated or analysed during the current study.

## Abbreviations

A/CS-12: 12-Item anorexia/cachexia scale
AHI: Apnea/Hypopnea Index
AHNS: American Head and Neck Society
ASCO: American Society of Clinical Oncology
DDW: Digestive Disease Week
EAT-10: Eating Assessment Tool-10
EORTC QLQ-C30: European Organization For Research And Treatment Of Cancer Core Quality of Life questionnaire
EORTC QLQ-CX24: EORTC cervical cancer questionnaire
EQ-5D-3L: European Quality of Life Five Dimension questionnaire
ERS: European Respiratory Society
ESMO: European Society for Medical Oncology
FACT-C: Functional Assessment of Cancer Therapy-Colorectal
FACT-E: Functional Assessment of Cancer Therapy-Esophageal
FACT-ECS: Functional Assessment of Cancer Therapy-Esophageal Cancer Subscale
FACT-G: Functional Assessment of Cancer Therapy-General
FIGO: International Federation of Gynecology and Obstetrics
HADS: Hospital Anxiety and Depression Scale
HRQoL: Health-related quality of life
HUS: Health Utility Score
ISPOR: Professional Society for Health Economics and Outcomes Research
MCED: Multi-cancer early detection
MCS: Mental Component Summary
MDASI: MD Anderson Symptom Inventory
NCCN DT: National Comprehensive Cancer Network Distress Thermometer
OR: Odds Ratio
PCS: Physical Component Summary
PICOS: Population, Intervention, Comparison, Outcomes, Study Design
PROMIS: Patient-Reported Outcomes Measurement Information System
PROMs: Patient reported outcome measures
PSQI: Pittsburgh Sleep Quality Index
PSSCAN-R: Revised Psychosocial Screen for Cancer
SCL-17: National Surgical Adjuvant Breast and Bowel Project (NSABP) Symptom Checklist
SF-12: Short-Form Survey-12
SF-36: Short-Form Survey-36
SF-8: Short-Form Survey-8
TOI: Trial Outcome Index
UICC: Union for International Cancer Control
USPSTF: US Preventive Services Task Force
